# The complete mitochondrial genome and phylogenetic analysis of *Omphalius rusticus* (Gastropoda, Tegulidae)

**DOI:** 10.1080/23802359.2020.1772146

**Published:** 2020-06-02

**Authors:** Shuai Mao, Chengrui Yan, Jie Bai, Yingying Ye, Jiji Li, Yahong Guo, Jiantong Feng

**Affiliations:** National Engineering Research Center for Marine Aquaculture, Marine Science and Technology College, Zhejiang Ocean University, Zhoushan, PR China

**Keywords:** *Omphalius rusticus*, mitochondria genome, phylogenetic analysis

## Abstract

The complete mitochondrial genome of the *Omphalius rusticus* has been determined. The complete genome is 18,067 bp and contained 13 protein-coding genes, two rRNA genes and 22 tRNA genes. The overall base composition is 33.48% (A), 33.52% (T), 15.58% (G) and 17.42% (C). The all start codon for 13 protein-coding genes is ATG and the most common termination codon is TAA. The phylogenetic tree showed that *O. rusticus* is most closely related to the *Tectus pyramis*. We suggest that this result will further supplement the genome information in mitochondria of the family Tegulidae and facilitate the study on population genetics.

*Omphalius rusticus* belongs to the family Tegulidae. It is widely distributed along the coast of China, South Korea (Sato-Okoshi et al. 2012), Japan (Fuji and Nomura 1990), and other western Pacific regions. The length of adult shells is between 10 and 20 mm. It is an edible perennial gastropod that can live for 5 years or more, and it is herbivorous. *O. rusticus* is dioecious and oviparous, and the spawning stage took place from July to September (Lee 2001). *O. rusticus* has been developed as a kind of sea food because of its high protein and low fat. To date, however, less research is relative to the sequence of the mitochondrial DNA genes of *O. rusticus.*

Herein, it is the first time to determine the complete mitochondrial genome of *O. rusticus*. This study will help to understand the phylogenetic status of genus *Omphalius* among the Class Gastropoda and Phylum Mollusca. The specimen of *O. rusticus* was collected in Penglai, Shandong Province, China (120.7°E, 37.8°N) and identified by morphology and deposited in Zhejiang Ocean University (accession number: OR20181021). The total DNA extraction utilized the salting-out method (Aljanabi and Martinez 1997) with the muscle. Then, total genomic DNA was diluted to a final concentration of 60–80 ng/µl in 1× TE buffer and stored at 4 °C. The genomic DNA was prepared in 400 bp paired-end libraries. The Illumina HiSeq X Ten platform was used to perform the high-throughput sequence. All the data were available and enumerated to the Microsoft oneDrive database (https://1drv.ms/w/s!ArF1Al5lLW_Vctd6wLfMq_cTw-E?e=5i4rMT).

The length of the complete mitochondrial genome of *O. rusticus* is 18,067 bp with the GenBank accession no. MT366560. The complete mitochondrial genome has 13 protein-coding genes, two ribosomal RNA genes, and 22 transfer RNA (tRNA) genes. The mitochondrial base composition is A 33.48%, T 33.52%, G 15.58%, and C 17.42%, with an obvious (A + T) % > (G + C) %. In 13 protein-coding genes, all of them start with ATG. For the stop codon, atp8 ends with TAG and nad3 ends with TGA, other 11 genes end with TAA. The 16S rRNA is 1560 bp between the tRNA^Leu1^ and tRNA^Val^, and the 12S rRNA is 1005 bp between the tRNA^Val^ and tRNA^Met^.

The phylogenetic relationship is estimated using the Neighbour-joining method (Schull 1979) in the program Phylip (Felsenstein 1989) based on 13 protein-coding genes of *O. rusticus* and other 11 species (Figure 1). It is showed that the phylogenetic relationship of *O. rusticus* is very close to the *Tectus pyramis*. Meanwhile, the phylogenetic relationship of *O. rusticus* is far away from *Perna perna* and *Perna viridis*. We expect the present results will further supplement the genome information in mitochondria of the family Tegulidae and facilitate the study on the taxonomy, population genetic structure, and phylogenetic relationships.

**Figure 1.**
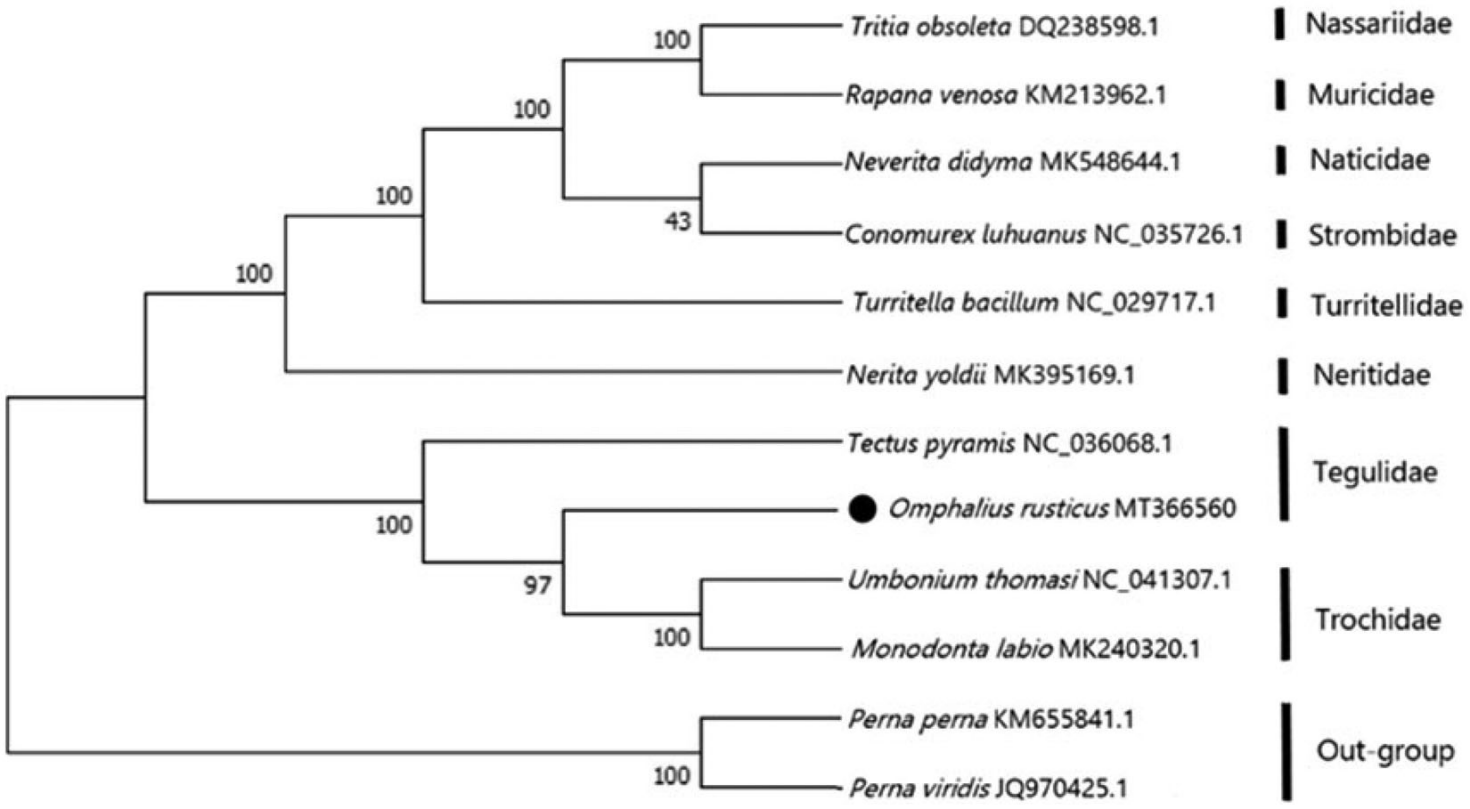
The NJ phylogenetic tree for *Omphalius rusticus* and other species based on 13 protein-coding genes. The black dot indicated the species in this study. The number at each node is the bootstrap probability. The number after the species name is the GenBank accession number.

## Data Availability

The data that support the findings of this study are openly available in Microsoft OneDrive at https://1drv.ms/w/s!ArF1Al5lLW_Vctd6wLfMq_cTw-E?e=5i4rMT; and in GenBank, reference number: MT366560.
